# Implementation of data access and use procedures in clinical data warehouses. A systematic review of literature and publicly available policies

**DOI:** 10.1186/s12911-020-01177-z

**Published:** 2020-07-11

**Authors:** Elena Pavlenko, Daniel Strech, Holger Langhof

**Affiliations:** 1 Charité – Universitätsmedizin Berlin, corporate member of Freie Universität Berlin, Humboldt-Universität zu Berlin, and Berlin Institute of Health, Berlin, Germany; 2 QUEST – Center for Transforming Biomedical Research, Charité – University Medicine, Berlin Institute of Health (BIH), Anna-Louisa-Karsch-Str. 2, 10178 Berlin, Germany; 3 Institute for History, Ethics and Philosophy of Medicine, Hannover Medical School (MHH), Hannover, Germany

**Keywords:** Clinical data warehouse, Data access and use, Data sharing, Ethics, Governance

## Abstract

**Background:**

The promises of improved health care and health research through data-intensive applications rely on a growing amount of health data. At the core of large-scale data integration efforts, clinical data warehouses (CDW) are also responsible for data governance, managing data access and (re)use. As the complexity of the data flow increases, greater transparency and standardization of criteria and procedures are required in order to maintain objective oversight and control. Therefore, the development of practice oriented and evidence-based policies is crucial. This study assessed the spectrum of data access and use criteria and procedures in clinical data warehouses governance internationally.

**Methods:**

We performed a systematic review of (a) the published scientific literature on CDW and (b) publicly available information on CDW data access, e.g., data access policies. A qualitative thematic analysis was applied to all included literature and policies.

**Results:**

Twenty-three scientific publications and one policy document were included in the final analysis. The qualitative analysis led to a final set of three main thematic categories: (1) requirements, including recipient requirements, reuse requirements, and formal requirements; (2) structures and processes, including review bodies and review values; and (3) access, including access limitations.

**Conclusions:**

The description of data access and use governance in the scientific literature is characterized by a high level of heterogeneity and ambiguity. In practice, this might limit the effective data sharing needed to fulfil the high expectations of data-intensive approaches in medical research and health care. The lack of publicly available information on access policies conflicts with ethical requirements linked to principles of transparency and accountability.

CDW should publicly disclose by whom and under which conditions data can be accessed, and provide designated governance structures and policies to increase transparency on data access. The results of this review may contribute to the development of practice-oriented minimal standards for the governance of data access, which could also result in a stronger harmonization, efficiency, and effectiveness of CDW.

## Background

Digitalization in health care and biomedical research has developed at a rapid pace. What previously consisted of error-prone and time-consuming manual documentation of information, often resulting in poorly structured data, has in large part been replaced by digitally supported or fully automatized processes. Despite persistent challenges, the widespread adoption of electronic health records (EHR) is one of many examples of this digital progress [1], adding to an ever-increasing amount of personal clinical data generated in routine health care delivery.

Much progress is expected from the reuse of such structured clinical data for secondary research purposes in a wide area of applications. Epidemiological and health economic research, comparative effectiveness research, health care quality improvement,learning health care systems (LHCS) and, last but not least, precision medicine rely on such promising developments [2–6]. Given an appropriate level of data quality, data-intensive research using big data analytics, machine learning, deep learning and artificial intelligence could be a true turning point for biomedical research and health care delivery [7].

One of the cornerstones of successful data reuse is an appropriate data infrastructure. However, due to differences in local, regional and national infrastructures, the information system landscape in the health care sector is largely characterized by heterogeneity [8]. Hospitals alone, for instance, require a broad spectrum of different IT solutions, such as electronic medical records, laboratory information systems, and individual solutions for clinical research (e.g., databases, registries) at the same time. In many cases, the interoperability of these systems is limited [9]. Thus, a major concern for the effective usage of clinical data even within one system is the often-fragmented data storage creating so called “data silos” [10, 11].

First developed for industry, data warehousing has been identified as a solution to overcome this siloed infrastructure [11]. Clinical data warehouses (CDW), more specifically, consolidate and integrate clinical data from various sources, such as health care data (e.g., from EHR), medical research data (e.g., from research biobanks or clinical trials) and patient-generated data (e.g., via mobile phones, smart-health applications, or wearables) [12]. When fully implemented, CDW allow for a broad and real-time analysis of data at the levels of individual patients and cohorts. The vast amount of data provided by CDW is thus seen as a key resource for data-intensive approaches such as research in precision medicine and quality improvement [12–15].

However, CDW are more than a mere technical infrastructure to integrate data. CDW hold responsibility over data stewardship [16], meaning the management and oversight of data, playing a crucial role in making stored clinical data accessible and (re)usable. With the multiplying promises of data-intensive research, the governance of data access and use gains an ethical dimension whose relevance is debated internationally [4, 17, 18].

An abundance of policies, regulations and guidelines addresses the importance of sharing data and making health data accessible for research purposes, thus calling for a transparent and sustainable data access governance [19]. For instance, the World Medical Association (WMA) in their Declaration of Taipei clearly demands, “Governance arrangements must include ( …) criteria and procedures concerning the access to and the sharing of health data” [20]. However, the WMA declaration is not legally binding and defines ethical standards on an abstract and theoretical level.

Challenges often arise when ethical standards are implemented in practice. In the case of biobanks, for instance, a recent investigation on sample and data access governance revealed significant shortcomings: although sample and data access policies are required, they are in most cases not publicly available and the criteria for access decisions outlined in the policies lack systematization and harmonization [21]. This is recognized as a threat to responsible and transparent (inter-)national collaboration and thus limits the prospects of networked biobanking [22].

Regarding data governance and stewardship, biobanks and CDW share common responsibilities. So far, the scientific literature offers little information about the actual practice of structuring and handling the governance of data access in CDW. A review by Holmes et al. on data warehouse governance for distributed research networks found that details on governance were sparse. However, their review was limited to publications from the U.S. before July 2013, and the authors expected more relevant publications to emerge in the years following their publication [23].

The aim of this study was to assess the spectrum of criteria and procedures applied in data access and use governance in CDW internationally.

## Methods

We performed a systematic review of (a) published scientific literature on CDW and (b) publicly available information on CDW data access. A protocol for this review was prepared using the PRISMA-P 2015 Checklist [24] and was preregistered and published within the Open Science Framework [25]. The methods of this study are presented in accordance with the Preferred Reporting Items for Systematic Reviews and Meta-Analyses (PRISMA) Statement where applicable [26].

### Search and selection

First, to review the scientific literature, the search engines PubMed, Web of Science, ACM Digital Library, CINAHL and IEEE Xplore were queried systematically. The PRESS checklist served to ensure the inclusion of essential elements in the search strategy [27]. The search terms were developed iteratively by piloting combinations of key words and MeSH terms in PubMed and the results were assessed for the inclusion of known representative literature. This process resulted in the combination of key words and MeSH terms of the search strategy presented in Table 1. The piloting was performed in October 2018, and the final search was conducted in November 2018.

**Table 1:** Search strategy

DATABASE	SEARCH TERM COMBINATION
***PUBLISHED SCIENTIFIC LITERATURE***	
PubMed	((((((((((warehouse [Title/Abstract]) OR data warehousing [MeSH Terms]) OR “data warehouse”) OR “data integration”) OR “data repository”) OR “Medical Informatics Applications”[Mesh]) OR Medical Record Linkage”[Mesh])) AND ((ethics [MeSH Terms]) OR governance))
Web of Science	TS = (clinical data warehouse OR clinical data repository OR data integration) AND TS = (ethics OR governance)
ACM Digital Library	content.ftsec:(+"clinical data warehouse”)
CINAHL	(TX ethics OR TX governance OR SU ethics OR SU governance) AND (TX data warehouse)
IEEE Xplore	(((clinical data warehouse) OR clinical data repository) OR data integration) AND ((ethics) OR governance)
Google Scholar	clinical data warehouse
***PUBLICLY AVAILABLE INFORMATION***	
Google	clinical data warehouse
Google	access policy AND data warehouse AND/OR clinical data

The retrieved scientific literature was imported into Endnote (Version X9.1) and screened for eligibility using RAYYAN [28]. RAYYAN is a freely available tool that facilitates the screening of literature (title and abstracts) for the purpose of systematic reviews. Two authors (EP, HL) independently screened all records and checked for eligibility and inclusion. In order to be included, scientific literature needed to explicitly report the development, implementation or maintenance of a CDW and provide information on the governance of data access and use. Further inclusion criteria were the language of the publication (English, German, or French) and the type of CDW. Only publications that described large-scale CDW (e.g., academic medical centers, larger hospitals) that process data collected from routine health care, and disease-general CDW (not, for example, CDW specific to diabetes) were included. We did not set any restriction based on the date of publication of the source.

After screening the first 100 references, we compared the initial results and discussed the appropriateness of the inclusion and exclusion criteria. In screening the full body of literature, 42 conflicting ratings occurred, all of which were resolved by discussion.

In addition, the search engine Google Scholar was consulted using the key search term elaborated in Table 1. The first 200 hits sorted by relevance were included. Finally, in a reference check, the references of all included publications were screened to check for additional literature that had not been captured by our search strategy.

Second, for the publicly available information, a web search was conducted via Google.de to identify the online presence of CDW. The first 100 hits sorted by relevance (by default) were screened for CDW websites (see Table 1). There, we searched for publicly available information on data access governance, such as linked access policy documents or access and use criteria addressed directly on the web pages. Further, we searched specifically for the web presences of the CDW of the included literature to check for online available policies.

In addition, Google.de was separately searched for “access policies” (see Table 1). All web searches were performed using Google.de in a private browsing mode of the Mozilla Firefox web browser, with all cookies deleted and all private accounts logged out of prior to the search.

### Analysis and synthesis

A qualitative thematic analysis was applied to all included literature [29]. Three main thematic categories were developed through an inductive strategy. The main categories were then applied to the literature, and the relevant text passages were coded using MAXQDA 2018 [30]. We sensitively extracted all the information potentially relevant to data access and use governance. In an iterative process using an inductive approach, we identified themes in our findings, which were clustered into subcategories and categories, dependent on their level of granularity. Through mutual agreement we reached consensus on the chosen terminology. Thus, we derived a matrix containing the spectrum of thematic categories and subcategories from the literature. Each subcategory of the last order is represented by at least one literary source (Supplement 3). As this review did not focus on the results of interventions, we did not anticipate the need for methods to minimize the risk of bias.

## Results

The systematic scientific literature search retrieved a total of 4249 references. After duplicates were removed, 4133 references were screened. Fifty-one references were then included in the full-text screening, 19 of which fulfilled the criteria and were included in the final analysis. Four additional publications were included via the Google Scholar searches. With the additional web search strategy applied in this study, we found 14 CDW web pages, none of which contained publicly available information on data access governance, such as linked access policy documents or access and use criteria (see supplement Table 1 for a list of the CDW web pages). We found one access policy that outlined the governance arrangements of CDWs and fulfilled our inclusion criteria, as determined by checking the web pages of the 23 CDWs of the publications included in the study. The reference check of all included publications did not yield additional literature or documents [11, 14, 15, 31–50]. One intermediate outcome of our study is therefore that only one out of 37 CDW (14 in website search, 27 from scientific literature) had a policy retrievable from their website.

**Fig. 1:**
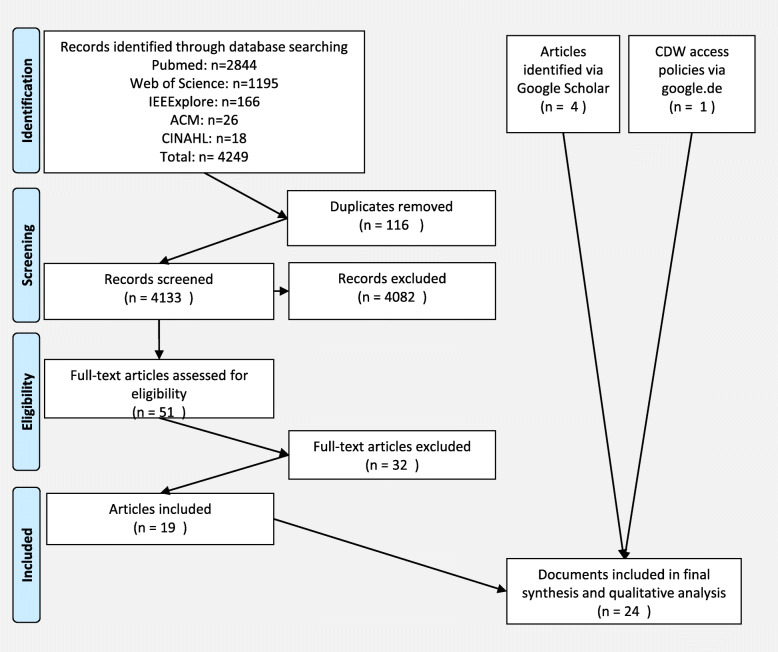
Data access review: Overview of the main categories and two subcategory levels

A final set of 24 documents (23 publications and one access policy) was included for the qualitative thematic analysis. See the PRISMA flow diagram in Fig. 1 for details. All included publications were in English. Twenty-two publications were published in peer-reviewed journals, and one was included in conference proceedings. See supplement Table 2 for details on all included documents.

The primary outcome of this systematic review is a matrix containing the qualitative spectrum of criteria and procedures applied in data access and use governance in CDW (see Table 2). The matrix is divided into three main categories: (1) requirements, including the subcategories recipient, reuse and formal requirements, (2) structures and procedures, including the subcategories review bodies and values, and (3) access, including the subcategory access limitations.

**Table 2:** Qualitative spectrum of criteria and procedures in data use and access governance in CDW

**MAIN CATEGORY**	**SUBCATEGORY**	**SUBCATEGORY**	**SUBCATEGORY**	**SUBCATEGORY**	
***REQUIREMENTS***					
	Recipient	Recipient categorization	Roles		
			Status	Authorized vs. non-authorized	
				Internal vs. external	
		Recipient background	Professional background	Academic staff	
				Healthcare professionals	
				Office staff	
				Researchers	
			Other background	Organizations	
				Patients	
				Students	
		Recipient qualifications	Human-subjects training		
		Recipient relation to CDW	Affiliation		
			Reciprocity		
	Reuse	Reuse purpose	Research purposes	General research	
				Clinical trials	
				Epidemiological studies	
				Feasibility check for research	
				Health services research	
				(Non-)human subject research	
				Observational studies	
				Personalized treatments	
			Health care purposes	Audit	
				Care management	
				Clinical care coordination	
				Clinical quality improvement	
				Facilitating organ, eye or tissue donation and transplantation	
				Public health reporting	
			Other reuse purposes	Grant application support	
				Marketing	
		Reuse setup	Clear documentation provided		
			Dissemination plan provided		
			Feasibility assessment provided		
			Funding provided		
			Process details provided		
		Reuse risk mitigation	Data protection		
			HIPAA compliance		
			IRB authorization		
			No contacting data subjects		
			No reidentification		
			No resharing without approval		
		Reuse values	Ethical soundness	Responsibleness	Compliance
					Privacy
					Responsible reuse
				Patient-centricity	Clinical relevance
					Patients first
				Non-competitiveness	Business issues
					Collaboration principles
					Non-profit
			Scientific soundness	Good research conduct	
				Innovation	
				Merits	
			Appropriateness	Appropriateness of requests	
				Appropriateness of results	
				Mission consistent with CDW	
				Reputation	
	Formal requirements	Data reuse documents	Data access/ sharing/ use agreement		
			Data collaboration agreement		
			Ethical agreements		
			Honorary/ substantive contract		
			Participation agreement		
			Term license agreement		
		General policies and regulations	Ethics	Consent	
				IRB approval	
			Laws	General laws	
				Specific laws	
			Other general policies and regulations	FAIR principles	
				Statewide policy guidance	
		Local policies and regulations	Information governance		
			Data access policies		
			Data identifying/ access/ sharing/ publishing protocols		
			Data management policies		
			Privacy/ security policies		
			Protocol review guidelines		
			Research policy		
			SOPs		
		Fees			
***STRUCTURES AND PROCEDURES***					
	Review bodies	Governance bodies	Board of directors		
			Clinical committee		
			Department head		
			Director of professional services		
			Executive board		
			IRB		
			Project review committee		
			Privacy board		
			Privacy and security committee		
			Policy advisory groups		
			Use and access committee		
			Working groups		
		Scientific review bodies	Academic staff		
			Data analysts		
			Health information research unit		
			Research committee		
			Scientific peer-review		
		Patient review bodies	Patient-led oversight committee		
	Review values	General values	Appropriate competency		
			Fine-grained control		
			Holistic governance		
		Reducing bias	Empirical evaluation		
			No conflict of interest		
			Objective review		
			Transparent review		
		Reducing investment	Effective review		
			Timely review		
		Managing competitiveness	Collaboration		
			Community before individual		
***ACCESS***					
	Access limitations	Limited data	Aggregated data set		
			De-identified data set		
			Limited data set		
		Limited location	Remote access		
			View only		
			Virtual access		
		Limited time			

All six subcategories are further split into more detailed subcategories addressing particular aspects relating to data access and use. See Table 2 for the full set of categories and subcategories. An expanded table giving exemplary quotes from the included literature for all subcategories is available as an online supplement (supplement Table 3).

The policy retrieved contributed substantially to our results [51]. What stood out was the clarity of definitions and terms in the document. However, while providing a detailed list for a data reuse “application”, it does not expand on what will ultimately be decisive for a data access and use decision: “( …) compliance with such other criteria as the Research Committee deems appropriate for the Research in question and the protection of Healthix. “(p.23) and “If deemed feasible, the application will then be presented to the Research Committee for review and final action, and such final action shall be communicated in writing to the requesting Researcher.” (p.25) [51]. In contrast, the scientific literature was lacking uniformity in terminology and precision overall. It also rarely provided clear indication of whether a certain aspect directly contributes to a data access and use decision.

**Fig. 2:**
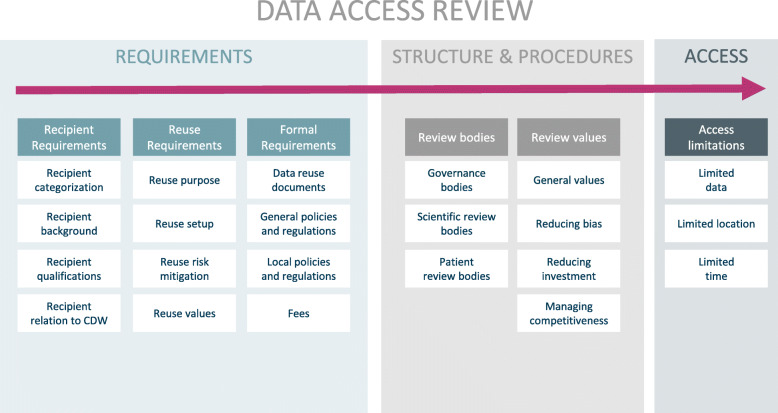
Systematic search flow diagram based on PRISMA

In the following, we present a brief definition of the themes to facilitate the understanding of the matrix. Figure 2 serves as an overview illustrating the relationship between requirements, structures and procedures, and access.

### Requirements

Under the main category “requirements”, we subsume all prerequisites linked to the data request. We distinguish between the requirements relating to the potential data recipient (recipient requirements), the requirements relating to the data reuse (reuse requirements) and the requirements relating to documents, policies or other formalities (formal requirements).

#### Recipient requirements

The recipient requirements include various definitions of potential data recipients, organized by their formal categorization, their background, their qualifications or their relation to the CDW.

*Recipient categorization*: Recipients can be categorized in different manners. On a general level, these categories can be divided into roles and status. Role represents a specific combination of attributes defined in detail by the respective CDW (e.g. background and qualifications combined). Status describes the presence or lack of one concrete attribute (e. g. authorized or not, internal or not).

*Recipient background*: On a more detailed level, potential data recipients are characterized by their background. This can be a specific professional background or another background relevant to the context of the CDW.

*Recipient qualifications*: Potential data recipients can be required to have certain qualifications in order to access the data, meaning that they have skills that enable them to use the data as required by the CDW.

*Recipient relation to CDW:* The potential data recipient’s relationship to the CDW might play a role in the data request. Some CDW might only agree to data access when a prior relation to the CDW or to the data provided to the CDW has been established.

#### Reuse requirements

The reuse requirements reflect different aspects of the planned data reuse that might influence access and use decisions.

##### Reuse purposes

Reuses can be described by their intended purpose, meaning the proposed objective of the data reuse. We intentionally excluded primary uses such as treatment and the closely related purposes of payment and insurance coverage review as presented in the policy document [51], as this review focuses on data reuse.

##### Reuse setup

Reuse setup describes organizational and practical level preparations for the data reuse that need to be provided for a data request.

##### Reuse risk mitigation

Reuse risk mitigation describes the potentially required commitments of the data reuser that serve to minimize the risk to the data subject stemming from the reuse of data.

##### Reuse values

Reuse values are requirements to the quality and integrity of the reuse request concerning ethical or scientific soundness, or appropriateness. *Ethical soundness* can be further subdivided into responsibleness, patient-centricity and non-competitiveness.

#### Formal requirements

Formal requirements are documents, policies and regulations which potential data recipients might need to sign, respect or agree to for a positive data access and use decision.

##### Data reuse documents

Data reuse documents describe mutual agreements and contracts specific to the reuse of the data.

##### General policies and regulations

This section describes fundamental laws and policies whose requirements are not specific to the CDW but more general.

##### Local policies and regulations

In contrast to the general policies and regulations, this subcategory comprises the policies and regulations where the content would be expected to vary strongly depending on the local particularities.

##### Fees

The data access and use decisions might also rely on the potential recipient’s agreement to paying a fee.

#### Structure and procedures

Under the main category “structure and procedures”, the governance structures and procedures are subsumed which need to be in place to provide for a review of the data request and the subsequent data access and use decision. They are divided into review bodies who are exercising the review, and review values underlying review procedures and decisions.

#### Review bodies

Review bodies reflect the implementation of designated governance bodies, which is commonplace in all assessed CDW. Here, we do not distinguish between boards, groups and individual positions, as the hierarchies and directions are not always transparent.

##### Governance bodies

This section comprises a variety of rather general governance bodies involved in the data reuse review.

##### Scientific review bodies

As a more specialized review body type, scientific review bodies describe those dealing with research questions.

##### Patient review bodies

Another type of specific review bodies are those where patients are involved in data access and use decisions.

#### Review values

Review bodies might follow certain review values when deciding data access and use requests which are subsumed in the following.

##### General values

General values describe values driving the CDW’s overall data access governance structure and procedures.

##### Reducing bias

This section encompasses the measures taken to reduce bias in the data request review.

##### Reducing investment

Reducing investments reflects aspects that save valuable resources, such as time and effort.

##### Managing competitiveness

Managing competitiveness describes an approach that places common interest over individual interest.

### Access

Under the main category “access”, we subsume data access which can be granted with varying limitations based on the previously described requirements or particularities of the CDW.

#### Access limitations

Access limitations can take on different forms and restrict either data itself, location or the access time.

##### Limited data

Data access can also be limited in itself, if only a specific part of the requested data is provided, or if it is only provided in a specific form that might be reduced in its level of information.

##### Limited location

Another possible limitation in access can be the location, where data can only be accessed from specific places.

##### Limited time

Some CDW might restrict the access to data to a certain timeframe or number of accesses.

## Discussion

This systematic review aimed to assess the spectrum of criteria and procedures that surround data access and use decisions in CDW. A core finding is the lack of concrete information available in the scientific literature and the lacking public accessibility of CDW data access policies.

### Focus of the current scientific discussion

While there is a solid corpus of scientific literature on the implementation and maintenance of CDW, the governance of data access is only marginally addressed. In many cases, the publications retrieved mention the relevance of data access and use without clarifying the details on the handling in practice. Many of the publications instead focus on technical aspects of data governance. Since the practical aspects of data access and use only become relevant when a significant amount of data is available for sharing, it is reasonable that CDW might approach technical issues first, which also require considerable effort and resources.

However, international recommendations on the ethics of health data sharing also emphasize the importance of procedural (e.g. independent review) and substantial aspects (e.g. criteria for data access decisions) of data access governance [20, 52]. The design and implementation of such sound governance arrangements for data access and use are nonetheless challenging [53].

Procedural aspects might be comparatively straightforward. Despite the heterogeneity of the denominations and of the composition of governance bodies, based on our study such procedural aspects seem to be addressed appropriately in the scientific literature.

Substantial aspects can be considered more demanding. The elaboration of concise access criteria satisfying all stakeholders and enabling all foreseen data reuses is a highly complex task. Considering the amount of public funding invested in the establishment of CDW, sharing details on the conceptualization and the access and use criteria themselves with the scientific community and the public is all the more important. This could significantly improve the efficiency of funding devoted to CDW.

### Accessibility of policies, and access and use criteria

In publications on transparent access and use governance, we would expect to find concrete and decisive criteria on the basis of which data access requests are weighted and judged. While the existence of more concrete policies is mentioned in the publications included, they could not be retrieved in our separate search except for one.

In the additional web search for publicly available access and use policy documents, our strategy was reduced to simpler search terms (see Table 1). A more refined search string could have potentially produced more policy documents. While this can be considered a limitation of our study, it also emulates how researchers and the public would most likely search for information on CDW.

To our knowledge, other than for biobanks [54] there are no public registries for CDW that would help access such information. It therefore appears that the access to those policies is limited, both by the CDW themselves and through a lack of appropriate search instruments. This is an important finding, as it reveals significant shortcomings in the compliance of CDW with the internationally agreed upon and accepted ethical standards of transparency, as required, for instance, by the WMA [20].

Further research could focus on possible relevant factors for this finding, for instance correlations of different types of CDW (e.g. population-based versus clinical cohorts) and the governance of data access and use. In addition, a systematic review of data sharing networks access and use governance could offer valuable results, especially when considering developing best practice examples.

### Transparency of criteria and its ethical value

Through our screening, we were able to extract potential criteria for decisions on data access and use. However, most of these criteria were not e xplicitly described as being decisive for a data request. Criteria applicable to the handling of data access requests are often provided in a rather unspecific and unstructured manner. They are not addressed in a separate designated section of the publications but are mentioned secondarily or need to be deduced from other information. Even the more explicit access and use criteria offer considerable leeway for interpretation and are therefore difficult to translate into practice. In addition, the denomination of requirements, structures and procedures varies highly between CDW and often lacks a more distinct definition. This can be detrimental to the harmonization and effectiveness of the CDW’s objectives.

The need for more clarity and standards on conditions for access and use must also be considered an ethical issue. While information transparency is not an ethical principle on its own, it can certainly become ethically relevant, for example when ethical principles such as informed consent or accountability depend on that information [55].

One could argue that individuals consenting to their data being stored and processed by a CDW rely on the CDW’s governance structures to execute the data reuse, first, according to their given consent, and second, with their best interests in mind beyond the consent. In their publication on the governance of biobanks, the German Ethics Council states that in order to compensate for the lack of precision at the time of consenting, donors should have the capacity to trace the governance of sample and data transfer. The specific purposes of reuse should therefore be publicly accessible in “a clear, generally intelligible and up-to-date account” [56]. This should be considered analogous for CDW. Transparency can therefore be considered a means to involve the data subjects and to give them a higher degree of data control. While some CDW embrace the direct involvement of patients in governance processes, for example in patient-led oversight committees, such measures are likely to improve transparency on a more individual level.

Particularly important, moreover, is the role of transparency as a requirement for setting up accountability mechanisms. Only clear conditions and attributions allow for the evaluation and execution of compliance with ethical and other norms. Ultimately, accountability also serves to increase public trust in data reuse [19].

Increased trust in CDW is also likely to have a positive effect on the future willingness to share data [57]. In contrast, the limited availability of information in the scientific literature and on CDW websites might prove to be detrimental. On a larger scale, this could weaken the potential of LHCS and other promising outcomes of secondary uses of data, such as precision medicine or comparative effectiveness studies, as they rely on the public’s investment in research.

### Return on public investment

Expectations placed on data-driven approaches in biomedical research and health care are high. Accordingly, increased efforts are being made internationally to develop and implement IT infrastructures that facilitate high-quality data access and use. In Germany, for instance, the Medical Informatics Initiative funding scheme was launched by the Federal Ministry of Research and Education (BMBF) in 2016 and aims to foster collaborative data use by investing 150 million Euros into the development of robust IT solutions [58].

This considerable allocation of funding, however, is generally based on the assumption of a return on public investment. Normative concepts and recommendations for the ethical conduct of data-driven approaches in medical research and health care have been developed and almost unanimously highlight the importance of data access [20, 59–61]. However, with an increasing amount of data requests for data-driven approaches, the case-by-case decisions as they appear to be reflected by our study will need to be replaced by a more automatized approach. In order to implement such automatization, the criteria and their weighting must be unambiguously clear.

Both, promoting scientific research and improving health care delivery, are very much in the public interest. While the public is supportive of the reuse of data for research purposes [35], it is the transparency of data access and use governance that might ensure their long-term support and trust through efficient reuse, informed consent, and accountability.

## Conclusion

To our knowledge, this is the first systematic review of data access and use in clinical data warehouses. The findings may stimulate further discussions about the appropriate governance of data access and use. Furthermore, the heterogeneity of mentioned substantial and procedural criteria that shall guide access and use decisions point to the need to develop practice-oriented standards. A certain degree of standardization may in the future contribute to more harmonization, efficiency, and effectiveness of data access and use governance. Involving all relevant stakeholders should be considered in the development and further improvement of data access and use policies in order to ensure acceptance and practice-oriented solutions. The results of this review, especially the qualitative spectrum of criteria and procedures in data access and use governance (Table 2) can serve as an evidence based starting point.

In the long run, more emphasis should be placed on the practice evaluation of CDW governance to assess compliance with ethical standards and to identify practical issues [62]. The latter, in turn, will be relevant for further policy development.

As digitalization and data-driven approaches in health research and health care are rapidly evolving, the current governance practices will require broader implementation, evaluation, and improvement to keep up with ongoing developments and challenges.

## Supplementary information

**Additional file 1: Supplement table 1.** List of CDW found in the Google search

**Additional file 2: Supplement table 2.** Included literature and policies

**Additional file 3: Supplement table 3.** Qualitative spectrum of criteria and procedures in data access and use governance in clinical data warehouses

## Data Availability

An extensive part of the data analyzed during this study is included in this published article and its supplementary files (Supplement Table 3). The raw dataset analyzed during the current study is not publicly available in full due to copyright restrictions but is available from the corresponding author on reasonable request.

## Abbreviations

BMBF: German Federal Ministry of Education and Research
CDW: Clinical data warehouse
EHR: Electronic health records
LHCS: Learning health care systems
PRISMA: Preferred Reporting Items for Systematic Reviews and Meta-Analyses
WMA: World Medical Association
