# Supplementary material for: Implementation of data access and use procedures in clinical data warehouses. A systematic review of literature and publicly available policies
Source: BMC Med Inform Decis Mak. 2020 Jul 11;20:157. doi: 10.1186/s12911-020-01177-z (PMC7353743; doi:10.1186/s12911-020-01177-z)
Supplement: Supplementary file 1 — Additional file 1: Supplement table 1. List of CDW found in the Google search [file 12911_2020_1177_MOESM1_ESM.pdf]

Supplement table 1 List of CDW found in the Google search

No.	CDW	WEBPAGE LINK	POLICY
1	University of Chicago Center for Research Informatics Clinical Research Data Warehouse	http://cri.uchicago.edu/crdw/	N/A
2	Stanford Medicine Research Informatics Center	http://med.stanford.edu/ric.html	N/A
3	UTHealth	https://sbmi.uth.edu/uth-big/clinical-data-warehouse/index.htm	N/A
4	University of Arizona Health Sciences	https://cb2.uahs.arizona.edu/clinical-data-warehouse	N/A
5	Emory Healthcare	http://it.emory.edu/ClinicalResearchData/index.html	N/A
6	UNC Health Care	https://tracs.unc.edu/index.php/services/informatics-and-data-science/cdw-h	N/A
7	University of New Mexico School of Medicine	https://hsc.unm.edu/research/ctsc/informatics/data-warehouse.html	N/A
8	Arkansas Clinical Data Repository	https://tri.uams.edu/tri-services/arkansas-clinical-data-repository-3/	N/A
9	University of Minnesota Clinical Data Repository	https://www.ctsi.umn.edu/researcher-resources/clinical-data-repository	N/A
10	Health Sciences South Carolina	https://www.healthsciencessc.org/tools-services/tool/clinical-data-warehouse/219	N/A
11	Vanderbilt University Medical Center	https://www.vumc.org/dbmi/research-data-warehousing	N/A
12	Rutgers Cancer Institute of New Jersey	https://gemini.cinj.rutgers.edu/projects/clinical-data-warehouse/	N/A
13	University of Illinois at Chicago	http://www.ccts.uic.edu/content/requesting-data-extracts-irb-requirements	N/A
14	Star Data Warehouse	https://ictr.johnshopkins.edu/programs_resources/programs-resources/informatics/epic-cogito-data-warehouse/	N/A

Based on the Google.de query “clinical data warehouse”, first 10 pages, sorted by relevance (default)
