# Supplementary material for: Implementation of data access and use procedures in clinical data warehouses. A systematic review of literature and publicly available policies
Source: BMC Med Inform Decis Mak. 2020 Jul 11;20:157. doi: 10.1186/s12911-020-01177-z (PMC7353743; doi:10.1186/s12911-020-01177-z)
Supplement: Supplementary file 2 — Additional file 2: Supplement table 2. Included literature and policies [file 12911_2020_1177_MOESM2_ESM.pdf]

Supplement table 2 Included literature and policies

No.	FIRST AUTHOR	YEAR OF PUBLICATION	JOURNAL	TITLE	POLICY ON WEB
1.	Bouzille	2017	Medinfo	Sharing Health Big Data for Research - A Design by Use Cases: The INSHARE Platform Approach	N/A
2.	Chute	2010	Am Med Inform Assoc	The Enterprise Data Trust at Mayo Clinic: a semantically integrated warehouse of biomedical data	N/A
3.	Des Jardins	2014	EGEMS	The Keys to Governance and Stakeholder Engagement: The Southeast Michigan Beacon Community Case Study	N/A
4.	Fleischmann	2014	EGEMS	The Visit-Data Warehouse: Enabling Novel Secondary Use of Health Information Exchange Data	"Healthix Security Policies and Procedures", https://healthix.org/who-we-are/healthix-policies/
5.	Foran	2017	Cancer Informatics	Roadmap to a Comprehensive Clinical Data Warehouse for Precision Medicine Applications in Oncology	N/A
6.	Ford	2009	BMC Health Services Research	The SAIL Databank: building a national architecture for e-health research and evaluation	N/A
7.	Grant	2006	International Journal of Medical Informatics	Integrating feedback from a clinical data warehouse into practice organisation	N/A
8.	Haarbrandt	2018	Methods Inf Med.	HiGHmed – An Open Platform Approach to Enhance Care and Research across Institutional Boundaries	N/A
9.	Hazlehurst	2015	International Journal of Medical Informatics	CER Hub: An informatics platform for conducting comparative effectiveness research using multi-institutional, heterogeneous, electronic clinical data	N/A
10.	Horvath	2010	Journal of Biomedical Informatics	The DEDUCE Guided Query tool: Providing simplified access to clinical data for research and quality improvement	N/A
11.	Jannot	2017	International Journal of Medical Informatics	The Georges Pompidou University Hospital Clinical Data Warehouse: A 8-years follow-up experience	N/A
12.	Laws	2014	EGEMS	The Community Health Applied Research Network (CHARN) Data Warehouse: a Resource for Patient-Centered Outcomes Research and Quality Improvement in Underserved, Safety Net Populations	N/A

13.	Lowe	2009	AMIA 2009 Symposium Proceedings	STRIDE – An Integrated Standards-Based Translational Research Informatics Platform	N/A
14.	Liu	2009	AMIA 2009 Symposium Proceedings	Toward a Fully De-identified Biomedical Information Warehouse	N/A
15.	Perera	2016	BMJ Open	Cohort profile of the South London and Maudsley NHS Foundation Trust Biomedical Research Centre (SLaM BRC) Case Register: current status and recent enhancement of an Electronic Mental Health Record-derived data resource	N/A
16.	Prasser	2018	Methods Inf Med.	Data Integration for Future Medicine (DIFUTURE)	N/A
17.	Prokosch	2018	Methods Inf Med	MIRACUM: Medical Informatics in Research and Care in University Medicine	N/A
18.	Ross	2014	EGEMS	The HMO Research Network Virtual Data Warehouse: A Public Data Model to Support Collaboration	N/A
19.	Stark	2010	Journal of Dental Education	Consortium for Oral Health-Related Informatics: Improving Dental Research, Education, and Treatment	N/A
20.	Turley	2016	EDM Forum	Leveraging a Statewide Clinical Data Warehouse to Expand Boundaries of the Learning Health System	N/A
21.	Van Eaton	2014	EGEMS	Achieving and Sustaining Automated Health Data Linkages for Learning Systems: Barriers and Solutions	N/A
22.	Walij	2014	Am Med Inform Assoc	BigMouth: a multi-institutional dental data repository	N/A
23.	Winter	2018	Methods Inf Med	Smart Medical Information Technology for Healthcare (SMITH)* Data Integration based on Interoperability Standards	N/A
