# Supplementary material for: Implementation of data access and use procedures in clinical data warehouses. A systematic review of literature and publicly available policies
Source: BMC Med Inform Decis Mak. 2020 Jul 11;20:157. doi: 10.1186/s12911-020-01177-z (PMC7353743; doi:10.1186/s12911-020-01177-z)
Supplement: Supplementary file 3 — Additional file 3: Supplement table 3. Qualitative spectrum of criteria and procedures in data access and use governance in clinical data warehouses [file 12911_2020_1177_MOESM3_ESM.pdf]

***Supplement table 3: Qualitative spectrum of criteria and procedures in data access and use governance in clinical data warehouses***

| Main category | Subcategory | Subcategory | Subcategory | Subcategory | Example quotes | Source |
|---|---|---|---|---|---|---|
| ***Requirements*** | | | | | | |
| | **Recipient requirements** | **Recipient categorization** | Roles | | When logging into the system, users must choose one of four user role types (Table 2) and provide the appropriate IRB protocol numbers that authenticate against the DUHS IRB database. | Horvath |
| | | | Status | Authorized vs. non-authorized | The same holds for authenticated users who have been granted data access. | Winter |
| | | | | Internal vs. external | Supported by service-oriented Data Transfer Units, selected representatives from multiple stakeholder groups, including physicians, researchers and data protection commissioners, decide transparently and objectively on data access applications from internal and external requestors. | Haarbrandt |
| | | **Recipient background** | Professional background | Academic staff | In order to allow end users (faculty, residents, students) to access the i2b2 web client, we used the InCommon federation (incommon.org) which provides a secure and trusted identity management system for member universities and other research institutions. | Walji |
| | | | | Healthcare professionals | Access to the CDW is provided to all HEGP healthcare professionals (e.g., clinicians, pharmacists, nurses, and researchers). | Jannot |
| | | | | Office staff | Additionally, as mentioned previously, SEMBC also facilitated regular physician and office staff user groups to share early lessons learned and to iterate on interventions. | Des Jardins |
| | | | | Researchers | Access to the CDW is provided to all HEGP healthcare professionals (e.g., clinicians, pharmacists, nurses, and researchers). | Jannot |
| | | | Other background | Organizations | This agreement is reciprocal, and these organizations must be accepted by current participants. | Turley |
| | | | | Patients | Third, the Trust Center is also responsible for handling various processes, such as consent withdrawal or providing patients with access to their data. | Prasser |
| | | | | Students | In order to allow end users (faculty, residents, students) to access the i2b2 web client, we used the InCommon federation (incommon.org) which provides a secure and trusted identity | Walji |

| | | | | | | |
|---|---|---|---|---|---|---|
| | | | | | management system for member universities and other research institutions. | |
| | | **Recipient qualifications** | Human-subjects training | | Appropriate human-subjects training is verified by the user's sponsoring institution. | Turley |
| | | **Recipient relation to CDW** | Affiliation | | A second requirement is an appropriate affiliation with SLaM for those accessing the data, most usually taking the form of an honorary or substantive contract, or a 'research passport', but also covered on occasions by appropriate between-institution legal agreements as directed by the SLaM Caldicott Guardian—the statutory office overseeing the use of patient information in the NHS. | Perera |
| | | | Reciprocity | | For any organization to access the data, they must also contribute to the repository. | Walij |
| | **Reuse requirements** | **Reuse purpose** | Research purposes | General research | It specifies to individual users the limitations of their use of the data for research purposes and the agreements they must adhere to, including not using the tool to try to identify patients or systems. | Turley |
| | | | | Clinical trials | HMORN VDW data are used for studies on a broad range of topics in health services research and epidemiology, in observational studies and clinical trials. | Ross |
| | | | | Epidemiological studies | Clinical epidemiology (studies on disease risk factors, prognosis and semiology) | Jannot |
| | | | | Feasibility check for research | Access by Healthix to De-Identified Data or Limited Data Sets to advise a third party that is designing a clinical trial or study, as to the feasibility of identifying sufficient potential subjects to meet the qualifying criteria being considered for the clinical trial or study (…) | Healthix |
| | | | | Health services research | Projects reusing CDW data have been classified according to 3 groups: • Clinical epidemiology (studies on disease risk factors, prognosis and semiology) • Health service research including evaluation of professional practice and decision-making support evaluation• Clinical research such as projects to support clinical trials recruitment and management | Jannot |
| | | | | (Non-)human subject research | Research exempt from IRB review (non-human-subjects research) [Fig.] | Horvath |
| | | | | Observational studies | HMORN VDW data are used for studies on a broad range of topics in health services research and epidemiology, in observational studies and clinical trials. | Ross |
| | | | | Personalized treatments | Data will be made available to researchers for cross-site (distributed) analyses and to physicians by providing | Prasser |

| | | | | | | |
|---|---|---|---|---|---|---|
| | | | | | innovative views on integrated data and decision support components for personalized treatments. | |
| | | | Health care purposes | Audit | Activity for projects using linked data sets held by CDLS is audited by the CDLS Safe Haven Officer, helping to ensure that the user's project requirements (eg, clinical research, surveillance, service improvement or audit) are met, and projects progress within the agreed policy and practice framework. | Perera |
| | | | | Care management | Level 1 Uses mean Treatment, Quality Improvement, Care Management, and Insurance Coverage Reviews. | Healthix |
| | | | | Clinical care coordination | The Healthix Participant Agreement, signed by all participating organizations, and the Healthix Privacy and Security Policy, approved by the Healthix Board of Directors, both provide for the sharing of clinical and encounter data across the HIE for clinical care coordination and quality assurance purposes, provided that the patient has given consent. | Fleischmann |
| | | | | Clinical quality improvement | This user-friendly data extraction system is envisioned as a multiple-tool environment that will expedite access to clinical data stored in the organizational data warehouse, supporting grant applications, research projects, and quality improvement (QI) activities | Horvath |
| | | | | Facilitating organ, eye or tissue donation and transplantation | Healthix will provide Organ Procurement Organizations with access to Protected Health Information without Affirmative Consent solely for the purposes of facilitating organ, eye or tissue donation and transplantation. | Healthix |
| | | | | Public health reporting | If a Data Supplier or Participant is permitted to disclose Protected Health Information to a government agency for purposes of public health (…) Healthix may make that disclosure on behalf of the Data Supplier or Participant without Affirmative Consent. | Healthix |
| | | | Other reuse purposes | Grant application support | This user-friendly data extraction system is envisioned as a multiple-tool environment that will expedite access to clinical data stored in the organizational data warehouse, supporting grant applications, research projects, and quality improvement (QI) activities | Horvath |
| | | | | Marketing | Level 2 Uses mean any uses of Protected Health Information other than Level 1 Uses, including but not limited to Payment, Research and Marketing. | Healthix |

| | | | | | |
|---|---|---|---|---|---|
| | | **Reuse setup** | Clear documentation provided | The medical records department based on the explicit documentation of the project and predetermined procedures manages the attribution of security level to individual data fields. | Grant |
| | | | Dissemination plan provided | CHC involvement in the approval of all data sharing activities, including manuscript review prior to publication, was a critical component of the DUAs. | Laws |
| | | | Feasibility assessment provided | Researchers who use this type of data sometimes incorrectly assume that the presence of variables in the data model implies that those variables are populated at every site over all time; users are advised to ensure the availability of the data they need in a study feasibility assessment. | Ross |
| | | | Funding provided | Inform researcher, proposal comes back to IRB, if funded (…) Proposal funded? [Fig.] | Laws |
| | | | Process details provided | Clear definition is required of which data, by whom, when (for what time period), where and why the data will be analysed and how the data will be published. | Grant |
| | | **Reuse risk mitigation** | Data protection | Upon first entry into the system and every 6 months afterwards, the user must agree to the terms of use: Protect data; do not share data outside of Duke without approval; and store data only on Duke computer drives. | Horvath |
| | | | HIPAA compliance | Activities included developing necessary policies and procedures governing use and protection of data, and HIPAA and Health Information Technology for Economic and Clinical Health (HITECH) Act protocols; (…) exercising due diligence in making sure SEMBC's vendors and participants are HIPAA compliant; and conducting training, among other activities. | Des Jardins |
| | | | IRB authorization | Project-specific IRB approvals are required prior to provisioning of any data set, and are provided by the IRB of record for the site in which research is being performed, most often the IRB of the requesting principal investigator. | Turley |
| | | | No contacting data subjects | Before any data is made accessible, applicants must sign a Data Use Agreement, where they agree in writing to adhere to the terms and conditions of usage (e.g. that the data are to be used solely for the requested and approved purpose and that the recipient must not try to identify or contact the data subjects). | Prasser |
| | | | No reidentification | Before any data is made accessible, applicants must sign a Data Use Agreement, where they agree in writing to adhere to the terms and conditions of usage (e.g. that the data are to be | Prasser |

| | | | | | | | |
|---|---|---|---|---|---|---|---|
| | | | | | | used solely for the requested and approved purpose and that the recipient must not try to identify or contact the data subjects). | |
| | | | No resharing without approval | | | Upon first entry into the system and every 6 months afterwards, the user must agree to the terms of use: Protect data; do not share data outside of Duke without approval; and store data only on Duke computer drives. | Horvath |
| | | **Reuse values** | Ethical soundness | Responsibleness | Compliance | Data users are required to sign an access agreement that they will behave responsibly and comply with all policy and security measures in place before they are provided with the user account. | Ford |
| | | | | | Privacy | Respecting the privacy of Participant and Patient data: The applicant shall commit to adhere to Healthix Policy, including Section 1.8.6 | Healthix |
| | | | | | Responsible reuse | For this to happen, further data transformations are carried out to control the risk of disclosure, and the data user signs an access agreement for responsible data utilisation, in accordance with the policies in place and the specifications of the IGRP to comply with Information Governance. | Ford |
| | | | | Patient-centricity | Clinical relevance | Clinical relevance, not mere technical feasibility, is the driver for our clinical use cases and design choices. | Haarbrandt |
| | | | | | Patients first | Patients first: All activities in HiGHmed are centered on the question what benefits patients most. | Haarbrandt |
| | | | | Non-competitiveness | Business issues | The DRRC reviews data set requests from the perspectives of the clinical systems, and the IRBs review requests from the perspective of research systems; this ensures that research regulations, ethical concerns, and competitive business issues are systematically addressed for each request. | Turley |
| | | | | | Collaboration principles | The governance structure is designed to ensure that OMOP adheres to its guiding principles of transparency (work products are released in the public domain), collaboration, and empirical evaluation. | Stang |
| | | | | | Non-profit | Access to De-Identified Data or a Limited Data Set via Healthix by an entity that is not eligible under Healthix Policy to be a Participant, for Research reviewed and approved or granted a waiver by an Institutional Review Board organized and operated in accordance with 45 C.F.R. §164, provided that: Such entity is a not-for-profit organization whose mission is consistent with the mission of Healthix and its Participants | Healthix |

| | | | | | | |
|---|---|---|---|---|---|---|
| | | | Scientific soundness | Good research conduct | The institutional IRBs have to guarantee that the protocols respect national legislation regarding the protection of patient privacy and various codes of ethics and good conduct of research. | Jannot |
| | | | | Innovation | The committee will then evaluate its merits and ensure that it has not been previously undertaken. | Stark |
| | | | | Merits | The committee will then evaluate its merits and ensure that it has not been previously undertaken. | Stark |
| | | | Appropriateness | Appropriateness of requests | A Collaboration Review System, including an independent Information Governance Review Panel, was established to assess the appropriateness of requests to use the data, and of project results before publication. | Ford |
| | | | | Appropriateness of results | A Collaboration Review System, including an independent Information Governance Review Panel, was established to assess the appropriateness of requests to use the data, and of project results before publication. | Ford |
| | | | | Mission consistent with CDW | Access to De-Identified Data or a Limited Data Set via Healthix by an entity that is not eligible under Healthix Policy to be a Participant, for Research reviewed and approved or granted a waiver by an Institutional Review Board organized and operated in accordance with 45 C.F.R. §164, provided that: Such entity is a not-for-profit organization whose mission is consistent with the mission of Healthix and its Participants (…) | Healthix |
| | | | | Reputation | Not compromising the reputation of Healthix or its Participants: the Research Committee shall determine that the study is not intended to reduce the good reputation of Healthix or any of its Participants | Healthix |
| | **Formal requirements** | **Data reuse documents** | Data access/ sharing/ use agreement | | Before any data is made accessible, applicants must sign a Data Use Agreement, where they agree in writing to adhere to the terms and conditions of usage (e.g. that the data are to be used solely for the requested and approved purpose and that the recipient must not try to identify or contact the data subjects). | Prasser |
| | | | Data collaboration agreement | | The CDW is an integrated clinical data research platform with a system of operation and use based on a comprehensive Data Collaboration Agreement (DCA). | Turley |
| | | | Ethical agreements | | It consists of identifying 10 basic requirements: platform and data provider information, institution information, study information, ethical agreements (coming from an independent IRB-like comity), Data Sharing Agreement (from | Bouzille |

| | | | | | | |
|---|---|---|---|---|---|---|
| | | | | | data providers who are involved in the study), approved users (external users and internal operators of the platform), authentication and access, data use, audit and accounting, patient rights, data segregation. | |
| | | | Honorary/ substantive contract | | A second requirement is an appropriate affiliation with SLaM for those accessing the data, most usually taking the form of an honorary or substantive contract, or a 'research passport', but also covered on occasions by appropriate between-institution legal agreements as directed by the SLaM Caldicott Guardian—the statutory office overseeing the use of patient information in the NHS. | Perera |
| | | | Participation agreement | | Participation Agreement means the agreement made by and between Healthix and each of its Participants, which sets forth the terms and conditions governing the operation of Healthix and the rights and responsibilities of the Participants and Healthix with respect to participation in Healthix. | Healthix |
| | | | Term license agreement | | CERTAIN executed a term license agreement with Caradigm for the provision of an Amalga instance for each participant hospital, and therefore Caradigm was acting as a service provider on behalf of UW and was obligated under the same consulting-services and business-associate agreements executed between UW and the participant hospital. | Van Eaton |
| | | **General policies and regulations** | Ethics | Consent | Based on a fine-grained use-and-access control mechanism, usage of medical data by individuals and organizations is strictly controlled, documented and requires authorization through the patient's consent. | Haarbrandt |
| | | | | IRB approval | Investigators can obtain identifiable data for research use after an Institutional Review Board (IRB) approval is obtained. | Liu |
| | | | Laws | General laws | Data will be imported into the DIC and be shared in strict accordance with data use agreements, applicable laws and informed consent. | Prasser |

| | | | | | | |
|---|---|---|---|---|---|---|
| | | | | Specific laws | Activities included developing necessary policies and procedures governing use and protection of data, and HIPAA and Health Information Technology for Economic and Clinical Health (HITECH) Act protocols; monitoring policies, procedures and protocols for needed enhancements; developing and executing data sharing agreements, intervention-level memorandums of understanding and other required agreements; exercising due diligence in making sure SEMBC's vendors and participants are HIPAA compliant; and conducting training, among other activities. | Des Jardins |
| | | | Other general policies and regulations | FAIR principles | By establishing a shared information governance structure between all participating clinical sites in accordance with the FAIR principles of data reuse [13], and by iteratively introducing an extensible and scalable software architecture, the translation between care provision and biomedical research will be enhanced. | Haarbrandt |
| | | | | Statewide policy guidance | Statewide Policy Guidance means the set of policies and procedures, including technical standards and SHIN-NY services and products, that are developed through the Statewide Collaboration Process and adopted by NYS DOH as provided in 10 N.Y.C.R.R. Section 300.3, including the statewide policy guidance incorporated by reference in subdivision (c) of that section. | Healthix |
| | | **Local policies and regulations** | Information governance | | For this to happen, further data transformations are carried out to control the risk of disclosure, and the data user signs an access agreement for responsible data utilisation, in accordance with the policies in place and the specifications of the IGRP to comply with Information Governance. | Ford |
| | | | Data access policies | | The work of this UAC will be guided by local Use & Access Policies (UAP), which have been defined at each of the MIRACUM partner sites and are closely aligned to the "Cornerstone Use & Access Policy" defined by the NSC Working Group on Data Sharing. | Prokosch |
| | | | Data identifying/ access/ sharing/ publishing protocols | | For the data warehouse, guidelines were defined to assure that CHCs reviewed and approved protocols for identifying data elements, for accessing and sharing data (within Nodes and with the DCC), and for publication. | Laws |
| | | | Data management policies | | Together with HIRU, the IGRP assesses each proposal for compliance with Information Governance and with a suite of | Ford |

| | | | | | |
|---|---|---|---|---|---|
| | | | | Standard Operating Procedures (SOPs) and data management policies. | |
| | | | Privacy/ security policies | The Healthix Participant Agreement, signed by all participating organizations, and the Healthix Privacy and Security Policy, approved by the Healthix Board of Directors, both provide for the sharing of clinical and encounter data across the HIE for clinical care coordination and quality assurance purposes, provided that the patient has given consent | Fleischmann |
| | | | Protocol review guidelines | For the data warehouse, guidelines were defined to assure that CHCs reviewed and approved protocols for identifying data elements, for accessing and sharing data (within Nodes and with the DCC), and for publication. | Laws |
| | | | Research policy | Compliance with Research policy: Healthix staff shall confirm that the proposal is consistent with Healthix Policy, before forwarding the proposal to the Research Committee. | Healthix |
| | | | SOPs | Together with HIRU, the IGRP assesses each proposal for compliance with Information Governance and with a suite of Standard Operating Procedures (SOPs) and data management policies. | Ford |
| | | **Fees** | | Healthix will establish a standard fee schedule to use Data for Research purposes, and the applicant will agree to pay such fees. | Healthix |
| ***Structures and procedures*** | | | | | |
| | **Review bodies** | **Governance bodies** | Board of directors | The Healthix Participant Agreement, signed by all participating organizations, and the Healthix Privacy and Security Policy, approved by the Healthix Board of Directors, both provide for the sharing of clinical and encounter data across the HIE for clinical care coordination and quality assurance purposes, provided that the patient has given consent | Fleischmann |
| | | | Clinical committee | Any uses of Protected Health Information for evaluating and improving operations shall be subject to prior consideration by a subcommittee (Data Use Subcommittee) including members of the Healthix Privacy and Security Committee, the Healthix Clinical Committee, and Healthix staff. | Healthix |
| | | | Department head | Approve: Coordinate with Dept Head/Provider, comments to Researcher, research may proceed | Laws |
| | | | Director of professional services | All data are encrypted on storage into the database and data access is only sanctioned on a project by project basis by the director of professional services supported with reference as necessary to the institutional ethics committee. | Grant |

| | | | | | |
|---|---|---|---|---|---|
| | | | Executive board | The 10-member executive board is chaired by the director of the FDA's Center for Drug Evaluation and Research, with membership drawn from academia, regulatory agencies, the pharmaceutical industry, data holders, patient advocacy groups, and health care providers (Appendix, available at www.annals.org). | Stang |
| | | | IRB | Project-specific IRB approvals are required prior to provisioning of any data set, and are provided by the IRB of record for the site in which research is being performed, most often the IRB of the requesting principal investigator. | Turley |
| | | | Project review committee | When a member of COHRI is interested in undertaking a research project utilizing the pooled database, he or she will submit a written proposal to the Project Review Committee. | Stark |
| | | | Privacy board | Privacy Board means a review body that meets the membership requirements of HIPAA and is established to act upon requests for a waiver or an alteration of the authorization requirements under HIPAA for uses and disclosures of Protected Health Information for a particular Research study | Healthix |
| | | | Privacy and security committee | Any uses of Protected Health Information for evaluating and improving operations shall be subject to prior consideration by a subcommittee (Data Use Subcommittee) including members of the Healthix Privacy and Security Committee, the Healthix Clinical Committee, and Healthix staff. | Healthix |
| | | | Policy advisory groups | The policy advisory groups provide expert advice regarding data quality, stewardship, IRB requirements and interactions, data use, privacy, and security. | Turley |
| | | | Use and access committee | Further, every MIRACUM partner has established a scientific Use & Access Committee (UAC), which is responsible for managing and evaluating all project proposals aiming at the use of DIC data either within the environment of the local MIRACUM site, but also for data sharing proposals within the MIRACUM consortium or even across all consortia. | Prokosch |
| | | | Working groups | WG2 "Data Sharing and Access, Consent and Quality Management" will deal with all aspects and regulations concerning patient consent, data sharing and access to the data, quality management, IT security and data protection, | Prokosch |

| | | | | | |
|---|---|---|---|---|---|
| | | **Scientific review bodies** | Academic staff | This manual review is carried out by the HIRU senior data analyst and the professor of public health, making use of the Office of National Statistics [32] guidance on disclosure control. | Ford |
| | | | Data analysts | This manual review is carried out by the HIRU senior data analyst and the professor of public health, making use of the Office of National Statistics [32] guidance on disclosure control. | Ford |
| | | | Health information research unit | Requests for data are assessed via a Collaboration Review System (CRS), which comprises review by the HIRU team and by an independent Information Governance Review Panel (IGRP). | Ford |
| | | | Research committee | Proposal comes to Research Committee | Laws |
| | | | Scientific peer-review | This is separate to the scientific peer-review process and review by a research ethics committee. | Ford |
| | | **Patient review bodies** | Patient-led oversight committee | Service user involvement is ensured in the decision-making process of approving projects working with linked data held by CDLS, and the patient-chaired CRIS Oversight Committee reviews and approves all projects using CRIS-linked data. | Perera |
| | **Review values** | **General values** | Appropriate competency | The committee shall (1) have members with varying backgrounds and appropriate professional competency as necessary to review the effect of the Research protocol on individuals' privacy rights and related interests; (…) | Healthix |
| | | | Fine-grained control | Therefore, in addition to fine-grained use-and-access control, patients will be able to view and obtain their health data through user-friendly tools (e.g. mobile apps, patient portals) similar to the Personal Electronic Health Record (PEHR) approach [14, 15]. | Haarbrandt |
| | | | Holistic governance | As well as risks due to data processing, levels of anonymisation, and possible disclosure, issues relating to responsible custodianship, control of the data and transparency would also be addressed to create a holistic governance framework. | Ford |
| | | **Reducing bias** | Empirical evaluation | The governance structure is designed to ensure that OMOP adheres to its guiding principles of transparency (work products are released in the public domain), collaboration, and empirical evaluation. | Strang |
| | | | No conflict of interest | The committee shall (…) (3) not have any member participating in a review of any project in which the member has a conflict of interest. | Healthix |

| | | | | | |
|---|---|---|---|---|---|
| | | | Objective review | Supported by service-oriented Data Transfer Units, selected representatives from multiple stakeholder groups, including physicians, researchers and data protection commissioners, decide transparently and objectively on data access applications from internal and external requestors. | Haarbrandt |
| | | | Transparent review | Supported by service-oriented Data Transfer Units, selected representatives from multiple stakeholder groups, including physicians, researchers and data protection commissioners, decide transparently and objectively on data access applications from internal and external requestors. | Haarbrandt |
| | | **Reducing investment** | Effective review | HSSC/HSHI oversee that process, assuring it is timely and effectively managed and, once approved, that the data provided are consistent with both the DRRC and IRB approvals and requirements. | Turley |
| | | | Timely review | HSSC/HSHI oversee that process, assuring it is timely and effectively managed and, once approved, that the data provided are consistent with both the DRRC and IRB approvals and requirements. | Turley |
| | | **Managing competitiveness** | Collaboration | The governance structure is designed to ensure that OMOP adheres to its guiding principles of transparency (work products are released in the public domain), collaboration, and empirical evaluation. | Strang |
| | | | Community before individual | For example, relative to governance generally, stakeholders agreed to a set of principles that included placing community goals before individual organizational goals when deploying SEMBC work | Des Jardins |
| ***Access*** | | | | | |
| | **Access limitations** | **Limited data** | Aggregated data set | For example, only coarsely aggregated race information was approved for V1 due to the potential for identifying patients falling into racial categories occurring with low frequency | Laws |
| | | | De-identified data set | Together, through this governance model, researchers and health systems are enabled both to use de-identified data and to establish a system to request and engage data and collaborators beyond one’s own system. | Turley |
| | | | Limited data set | “LDS” (data shareable as a Limited Data Set for specific research purposes under HIPAA) | Hazelhurst |
| | | **Limited location** | Remote access | In order to facilitate this, a dedicated office suite was set up in SLaM premises, the ‘BRC Nucleus’ to accommodate staff and visitors accessing Case Register data, although remote access, with appropriate security, is also possible. | Perera |

| | | | | | |
|---|---|---|---|---|---|
| | | | View only | Can export a limited dataset; PHI may be available on-screen only | Horvath |
| | | | Virtual access | Virtual Data Analysis Platform | Prasser |
| | | **Limited time** | | Delivery of the information can be arranged in combination of several forms, including (1) 1-time data download, (2) limited-access data mart browsing capability including allowed data elements, (3) limited-time hypothesis-generating query capability over specified data points, and (4) live, online reports that can be filtered and aggregated by user. | Foran |
